# Incidence of low-level viremia and its impact on virologic failure among people living with HIV who started an integrase strand transfer inhibitors: a longitudinal cohort study

**DOI:** 10.1186/s12879-023-08906-5

**Published:** 2024-01-02

**Authors:** Xiaojie Lao, Hanxi Zhang, Meiju Deng, Qun Li, Qing Xiao, Lin He, Liying Ma, Aqian Song, Xuelei Liang, Fengting Yu, Hongxin Zhao, Fujie Zhang

**Affiliations:** 1 Department of Infectious Disease, Beijing Ditan Hospital Capital Medical University, Beijing, 100015 China; 2 WHO Collaborating Centre for Comprehensive Management of HIV Treatment and Care, Beijing Ditan Hospital Capital Medical University, Beijing, 100015 China; 3 Clinical Center for HIV/AIDS, Beijing Ditan Hospital Capital Medical University, Beijing, 100015 China; 4 State Key Laboratory of Infectious Disease Prevention and Control, National Center for AIDS/STD Control and Prevention, Chinese Center for Disease Control and Prevention, Beijing, 102206 China; 5 Department of Gastroenterology, Beijing Ditan Hospital Capital Medical University, Beijing, 100015 China

**Keywords:** Antiretroviral therapy, Viral load, Low-level viremia, Virologic failure, HIV, People living with HIV, Integrase strand transfer inhibitors, INSTIs, Risk factors

## Abstract

**Background:**

Low-level viremia (LLV) has been identified as a potential precursor to virologic failure (VF), yet its clinical implications, particularly within the context of Integrase Strand Transfer Inhibitors (INSTIs)-based regimens, remain insufficiently explored. The study aimed to investigate the relationship between LLV and VF within ART-naïve patients on INSTIs-based regimens in China.

**Methods:**

A longitudinal cohort study was conducted with ART-naïve patients aged ≥ 18 years at Beijing Ditan Hospital, under the Chinese National Free Antiretroviral Treatment Program (NFATP). The LLV was defined as a viral load (VL) ranging from 50 to 199 copies/mL after six months of ART initiation, and VF as a VL ≥ 200 copies/mL. Sensitive analyses were also performed, defining LLV as 50–999 copies/mL and VF as exceeding 1000 copies/mL. Multivariate logistic regression, Kaplan-Meier (KM) curve, and Generalized Estimating Equation (GEE) models were used to evaluate the risk factors associated with LLV and VF events.

**Results:**

The study involved 830 ART-naïve patients, comprising 600 in the INSTIs group and 230 in the protease inhibitors (PIs) group. LLV events were observed in 10.4% of patients on PIs-based regimens and and 3.2% on INSTIs-based regimens (*P* < 0.001). INSTIs-based regimens demonstrated a protective effect against LLV events (aHR = 0.27, 95% CI 0.137–0.532). VF events occurred in 10.9% of patients on PIs-based regimens and 2.0% on INSTIs-based regimens, respectively (*P* < 0.001). The occurrence of LLV events significantly increased the risk of VF by 123.5% (95% CI 7.5%-364.4%), while the integrase inhibitors were associated with a 76.9% (95% CI 59.1%-86.9%) reduction in VF risk.

**Conclusion:**

Our findings indicate that INSTIs-based regimens are critical protective factors against LLV and subsequent VF. These results underscore the importance of HIV viral load monitoring to ensuring effective treatment outcomes, highlighting the necessity for prompt and precise monitoring to refine HIV treatment methodologies.

## Introduction

Globally, HIV remains a major public health burden, with approximately 39 million people live with HIV(PLWH), predominantly in sub-Saharan Africa and Southeast Asia [1]. Despite progress has been made in reducing new infections and AIDS-related deaths [2], challenges such as improving viral suppression rates and reducing drug resistance persist [3, 4]. Achieving effective viral suppression remaine crucial in the global fight against HIV to achieve UNAIDS targets of “95-95-95”. Integrase Strand Transfer Inhibitors (INSTIs) played a pivotal role in sustained viral suppression, which disrupted the viral life cycle by inhibiting the integrase enzyme, leading to rapid viral load reduction [5]. Recent studies have emphasized the efficacy of second-generation INSTIs like bictegravir and dolutegravir in maintaining persistent and undetectable viral loads [6, 7]. Nonetheless, even with INSTIs-based regimens, some individuals still undergo low-level viremia (LLV) despite achieving virological suppression [8].

The prevalence of LLV, ranging from 7% to 22%, varies based on detection thresholds, prescribed medications, and studied populations [8–13]. An Austrian cohort study found that PLWH on sustained ART with protease inhibitors (PIs)-based regimens had higher occurrences of LLV compared to those on NNRTIs/INSTIs-based regimens [14]. However, in a Taiwanese study of virally suppressed PLWH, the likelihood of manifesting LLV was similar between individuals transitioning to dolutegravir-based treatment and those persisting with PIs-based regimens [15]. This inconsistency in the data highlights a deficiency in our understanding of the prognostic significance of LLV in PLWH on INSTI-based first-line antiretroviral therapy (ART). Further investigations are warranted to elucidate the implications of LLV for long-term virological control during INSTI-based first-line ART.

Our previous study, along with studies from the US and European Cohorts, have consistently demonstrated an association between LLV and virologic failure (VF) in individuals with HIV [12, 16, 17]. However, the relationship between INSTIs-based regimens remains inconclusive. Studies from France and Taiwan have reported inconsistent findings. The French cohort demonstrated a significant correlation between LLV and VF in the context of first-line INSTIs-based regimens in ART-naïve patients [8], While the Taiwanese study compared LLV occurrences among patients receiving PIs, dolutegravir, or bictegravir as maintenance therapy after achieving viral suppression through previous treatment, reported low LLV rates and found no significant difference between LLV and VF [15, 18]. Given the international guidelines now advocate for INSTIs as first-line regimens [19–22], more evidence is needed to explore the potential implications of LLV on subsequent virological control, especially for patients receiving INSTIs-based regimens as their initial ART.

The objective of this study was to compare the incidence of LLV in treatment-naïve PLWH receiving INSTIs-based antiretroviral regimens versus those on PIs-based antiretroviral regimens and to estimate the subsequent risk of virological failure within a large longitudinal cohort in China. Our study aims to explore the long-term virological outcomes and potential risks associated with LLV in patients starting ART with INSTIs-based regimens.

## Methods

### Study population

This longitudinal cohort study was conducted by the Chinese National Free Antiretroviral Treatment Program (NFATP), the largest HIV Program in China, which provides free ART drug and laboratory monitoring in HIV care. We included ART-naïve patients aged ≥ 18 years old enrolled in the NFATP cohort in Beijing Ditan Hospital from July 2003 to July 2023. Patients with less than six months of antiretroviral treatment, missing viral load data atat baseline or during the follow-up period, and initial with NNRTIs-based regimen were excluded from the study.

### Outcome definitions

The viral load was routinely measured at 0, 6, and 12 months of the first year and thereafter once a year after the ART initiation. Additional viral load measurements were conducted at the clinician’s discretion in cases of VF and LLV. The baseline was defined as the demographic and clinical characteristics for assessing treatment outcomes before the ART initiation, especially in viral load measurement. This measurement served as a reference point against which subsequent viral load readings are compared to assess the efficacy of the treatment. In line with current guidelines, the viral suppression was defined as VL < 50 copies/mL after receiving 6 months of continuous ART, which is the detection limit of most modern ultrasensitive HIV viral load tests.

The LLV was defined as the VL ≥ 50 to ≤199 copies/mL after viral suppression or after six months of ART initiation, while the VF was defined as the VL ≥ 200 copies/mL after viral suppression or after six months of ART initiation. Multiple VL measurements between 50 and 199 copies/mL in the same year were considered as a single event of LLV. Multiple VL measurements between 50 and 199 copies/mL separated by at least one VL below 50 copies/mL were considered distinct events of LLV [20]. According to the WHO guidelines [22], we also performed a sensitive analysis with a broad level of LLV (defined as 50–999 copies/mL) as an alternative definition of LLV, while the VF was defined as ≥ 1000. copies/mL. Further, we also employ the more detailed virological definitions of intermittent low-level viremia (iLLV) and persistent low-level viremia (pLLV) to characterize the specific states of low-level viremia within our study cohort. The iLLV is defined as a single detection of low-level viremia, often referred to as a viral “blip”. In contrast, the pLLV is characterized by the detection of low-level viremia in at least two consecutive measurements.

### Statistical analysis

Continuous variables were described using median (interquartile range, IQR), and categorical variables were described using frequency (percentage). The χ2 or two-tailed Fisher’s exact test was used to compare categorical variables. The incidence rate of developing VF events over time was described by the Kaplan-Meier (KM) curve between PLWH receiving INSTIs-based regimen and PIs-based regimen. The log-rank test was utilized to statistically compare the differences in these survival curves between the two groups. The univariate and multivariate logistic regression model was performed to evaluate the protective effect of the ART regimen related to the occurrence of LLV events. Due to the low incidence of persistent low-level viremia (pLLV), our survival analysis and generalized linear regression models focused on the overall incidence of LLV events, without distinguishing between iLLV and pLLV events.

The generalized estimating equation (GEE) model was constructed to analyze the association between LLV events and the outcome of VF. The LLV events, including both intermittent (iLLV) and persistent (pLLV) occurrences, were treated as repeated measures within the GEE model. This approach allowed for the analysis of longitudinal viral load data from each patient, capturing the temporal correlations between successive LLV events and their potential impact on VF outcomes. All models were adjusted by sex, age, transmission routes, time from diagnosis to treatment, CD4 + T cell counts at ART initiation, and viral load at ART initiation. All statistical analyses were performed using SAS software version 9.4 (SAS Institute Inc, Cary, NC), and two-sided *P*-values were reported. The statistical visualization was performed by R version 4.2.0 with the package ggplot2.

## Results

### Participant characteristics

**Fig. 1:**
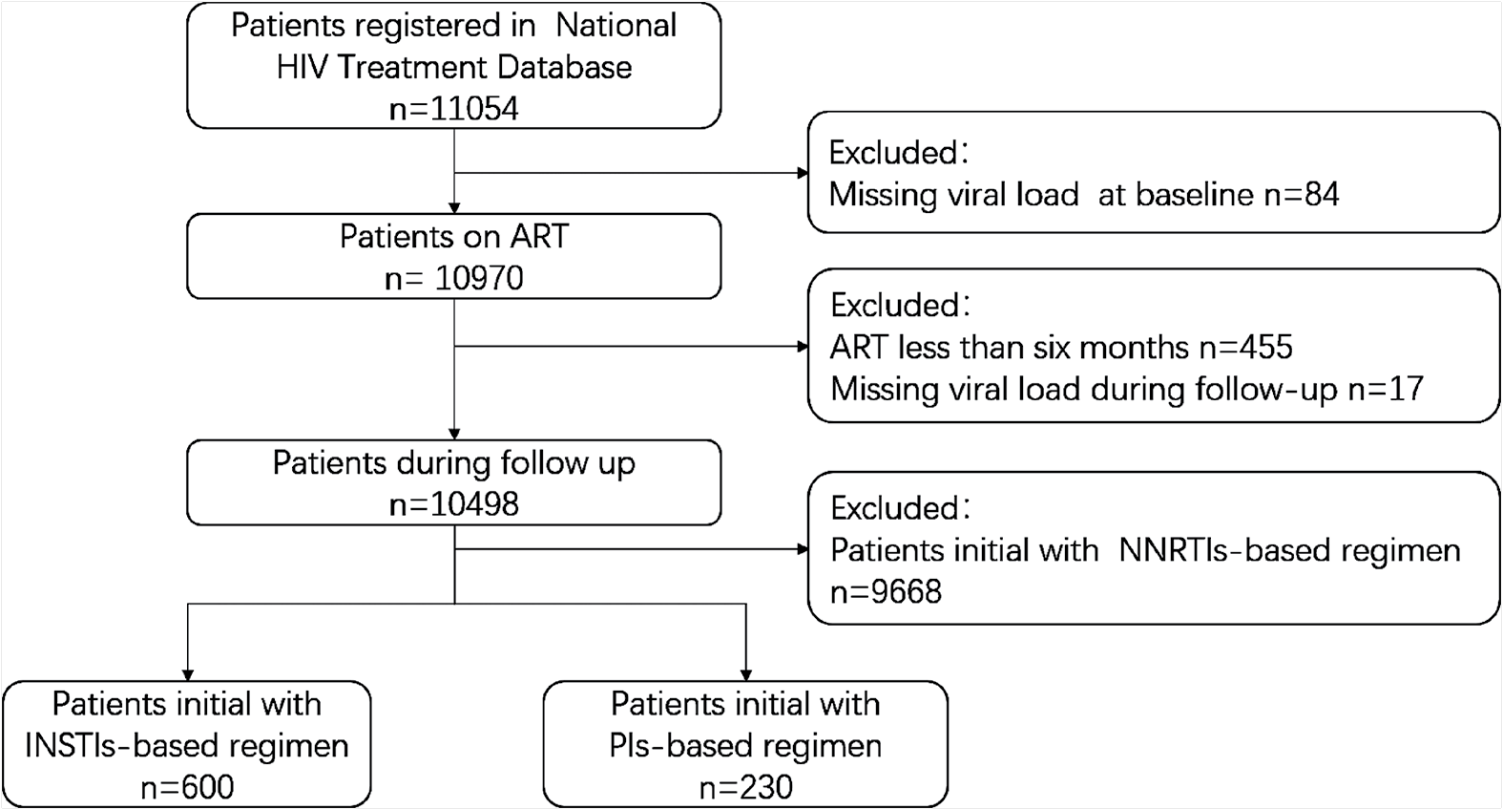
Study flow chart

Overall, a total of 830 people living with HIV-1 were included in the study (Fig. 1). Among them, 600 initially received the INSTIs-based regimen, while 230 started with the PIs-based regimen. The study population was predominantly male (91.2%) and mainly infected by homosexual sexual transmission (74.5%). The median age was 31.5 years old at ART initiation, the median CD4 + T cell count was 312 cells/uL (IQR 189–487), and the median VL was 11,400 copies/mL (41–70,597). Detailed distributions are presented in Table 1.

Figure 1 legend: This figure described the patient inclusion and exclusion criteria in our study, sourced from an extensive HIV treatment database comprising 11,054 individuals. A total of 830 patients were eligible for final analysis, comprising 600 on the initial INSTIs-based regimen and 230 on the initial PIs-based regimen. Abbreviations: ART: Antiretroviral therapy; INSTIs: Integrase Strand Transfer Inhibitors; PIs: Protease inhibitors; NNRTIs: Non-Nucleoside Reverse Transcriptase Inhibitors.

**Table 1:** Demographic and clinical characteristics

		INSTIs group	PIs group	Total
		(n = 600)	(n = 230)	(n = 830)
Sex, n (%)	Male	576 (96)	181 (78.7)	757 (91.2)
	Female	24 (4)	49 (21.3)	73 (8.8)
Age at ART initiation, years old, n (%)	18–25	124 (20.7)	71 (30.9)	195 (23.5)
	26–45	412 (68.7)	130 (56.5)	542 (65.3)
	> 45	64 (10.7)	29 (12.6)	93 (11.2)
	Median (IQR)	31.5 (27.2, 37.6)	30.8 (25.1, 39.9)	31.5 (26.9, 38.2)
Transmission, n (%)	Other	118 (19.7)	94 (40.9)	212 (25.5)
	Homosexual transmission	482 (80.3)	136 (59.1)	618 (74.5)
Time from diagnosis to treatment, month, n (%)	> 1	212 (35.3)	110 (47.8)	322 (38.8)
	≤ 1	388 (64.7)	120 (52.2)	508 (61.2)
Calendar time at ART initiation, n (%)	2003–2010	0 (0)	6 (2.6)	6 (0.7)
	2011–2017	3 (0.5)	150 (65.2)	153 (18.4)
	2018–2022	597 (99.5)	74 (32.2)	671 (80.8)
CD4 count at baseline, cells/uL, n (%)	≤ 200	141 (23.5)	87 (37.8)	228 (27.5)
	> 200	459 (76.5)	143 (62.2)	602 (72.5)
	Median (IQR)	344 (207, 518)	256 (101, 420.5)	312 (189, 487)
Viral load at baseline, copies/mL, n (%)	≥ 100,000	109 (18.2)	58 (25.2)	167 (20.1)
	< 100,000	491 (81.8)	172 (74.8)	663 (79.9)
	Median (IQR)	11,304 (52.5, 61907.5)	11836.5 (35.5, 102380.5)	11,400 (41, 70,597)

### Incidence rates of LLV

During the observation period, our study found 47 LLV events (defined as 50–199 copies/mL) among 43/830 individuals with an overall incidence rate of 5.2%. Within the INSTIs group, LLV occurred in 3.2% (19/600) of individuals, while a notably higher incidence of 10.4% (24/230) was observed in the PIs group (*P* < 0.001). When examining iLLV events, the INSTIs group presented a 3.0% incidence (18/600), compared to 9.6% (22/230) in the PIs group (*P* < 0.000). For pLLV, the incidence were lower, with 0.2% (1/600) in the INSTIs group and 1.3% (3/230) in the PIs group, though this difference was not statistically significant (*P* = 0.119). Notably, within the PIs group, there was one individual with a pLLV event following iLLV therapy. The overall median viral load of all LLV events in the study was 90 copies/mL (IQR 56–122). For the PI group, the median was 101 copies/mL (IQR 59–127.5), while the IN group had a median of 78 copies/mL (IQR 54.75–116) (*P* = 0.317).

In defining LLV as 50–999 copies/ml, 60 LLV events were recorded among 53/830 individuals (6.4% of the study population), with 3.8% (23/600) individuals occurring in the INSTIs group and 13.0% (30/230) individuals in the PIs group (*P* < 0.001). For iLLV, the incidence in the INSTIs group was 3.7% (22/600), compared to 12.2%(28/230) in the PIs group (*P* < 0.001). For pLLV, the occurrence was 0.2% (1/600) in the INSTIs group and 2.2% (5/230) in the PIs group (*P* = 0.009). Notably, within the PIs group, there were individuals with sequential LLV events, with two cases transitioning from iLLV to pLLV and one case from pLLV to iLLV. The median viral load across all LLV events was 133 (IQR 113–164.5) copies/ml. Specifically, in the INSTIs group, the median viral load was 95.5 (IQR 56.5–95.5) copies/ml, compared to 117.5 (IQR 66.75–220.75) copies/ml in the PIs group (*P* = 0.172).

### Factors associated with LLV

The univariate and multivariate logistic regression model was applied to investigate the identified factors associated with the occurrence of LLV events (Table 2). The INSTIs-based regimen was the protective factor associated with the occurrence of LLV events (defined as 50 to 199 copies/mL) with an adjusted hazard ratio (aHR) of 0.27 (95% CI 0.137–0.532) compared to the PIs-based regimen (*P* < 0.001). For the viral load at baseline, those with a viral load of more than 100,000 copies/mL had an aHR of 2.07 (95% CI 1.027–4.171) compared to those with a viral load of less than 100,000 copies/mL (*P* = 0.042). For CD4 + T cell count at baseline, comparison with CD4 + T cell count > 200 cells/µL, individuals with a count ≤ 200 cells/µL had a hazard ratio (HR) of 2.1 (95% CI 1.058–4.167) (*P* = 0.034). In the logistic model for LLV events, defined as 50 to 999 copies/mL, similar results were observed, indicating that INSTIs still exerted a protective effect (Table 3).

**Table 2:** Logistic regression analysis for risk factors associated with LLV events (defined as 50–199 copies/ml)

Risk factors	HR (95% CI)	*P-value*	aHR (95% CI)	*P-value*
Male (vs. Female)	0.937 (0.325, 2.7)	0.904	1.098 (0.304, 3.973)	0.887
Age > 25 (vs. < 25 years)	1.261 (0.486, 3.272)	0.634	1.503 (0.553, 4.086)	0.424
Homosexual Transmission (vs. other)	0.998 (0.494, 2.016)	0.995	1.282 (0.539, 3.049)	0.574
Time from diagnosis to treatment > 1 (vs. ≤ 1 months)	1.679 (0.849, 3.319)	0.136	1.869 (0.909, 3.844)	0.089
CD4 + T cell count at baseline ≤ 200 (vs. > 200 cells/µL)	3.265 (1.756, 6.069)	< 0.001	2.1 (1.058, 4.167)	0.034
Viral load at baseline > 100,000 (vs. ≤ 100,000 copies/mL)	3.418 (1.824, 6.404)	< 0.001	2.07 (1.027, 4.171)	0.042
INSTIs-based regimen (vs. PIs-based regimen)	0.281 (0.151, 0.524)	< 0.001	0.27 (0.137, 0.532)	< 0.001

**Table 3:** Logistic regression analysis for risk factors associated with LLV events (defined as 50–999 copies/ml)

Risk factors	HR (95% CI)	*P-value*	aHR (95% CI)	*P-value*
Male (vs. Female)	1.353 (0.558, 3.281)	0.504	1.152 (0.376, 3.526)	0.804
Age > 25 (vs. < 25 years)	0.918 (0.421, 2)	0.829	1.115 (0.489, 2.542)	0.797
Homosexual Transmission (vs. other)	0.953 (0.507, 1.792)	0.880	1.334 (0.596, 2.986)	0.483
Time from diagnosis to treatment > 1 (vs.≤ 1 months)	1.655 (0.895, 3.06)	0.108	1.835 (0.958, 3.515)	0.067
CD4 + T cell count at baseline ≤ 200 (vs. > 200 cells/µL)	2.741 (1.563, 4.808)	< 0.001	1.792 (0.96, 3.348)	0.067
Viral load at baseline > 100,000 (vs. ≤100,000 copies/mL)	3.093 (1.74, 5.499)	< 0.001	2.063 (1.082, 3.935)	0.028
INSTIs-based regimen (vs. PIs-based regimen)	0.266 (0.151, 0.468)	< 0.001	0.262 (0.141, 0.485)	< 0.001

### Factors associated with VF

**Fig. 2:**
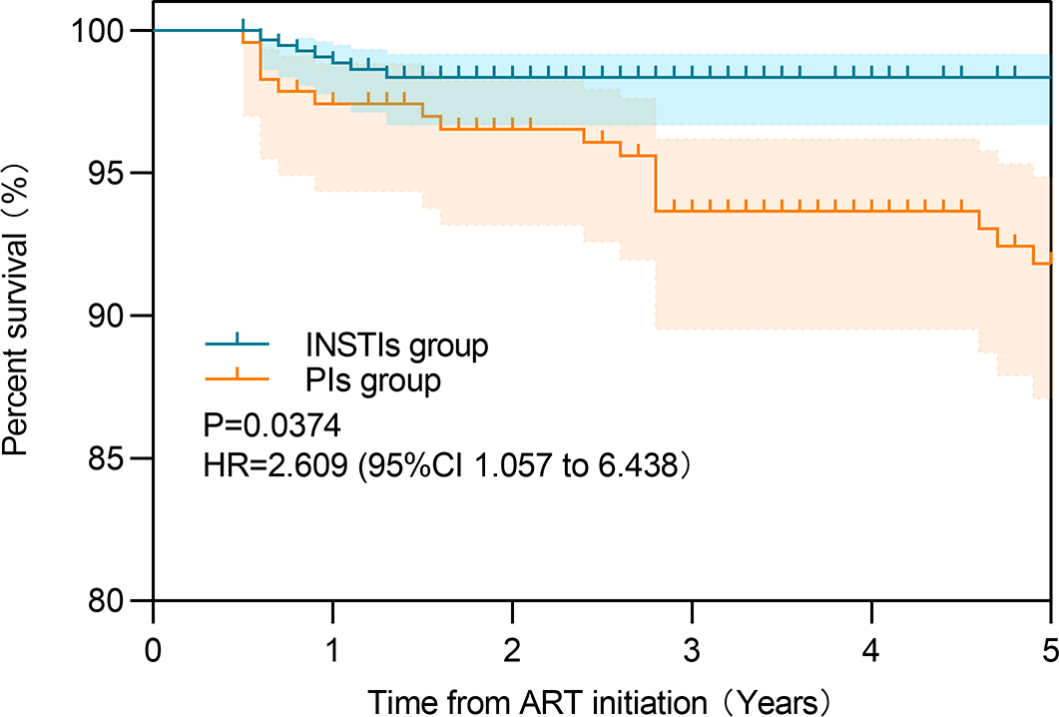
Kaplan-meier curves for virological failure (defined as ≥ 200 copies/mL) among people living with HIV treated with INSTIs-based regimen and PIs-based regimen

Based on the patient records, virological failure (VF) events, defined as viral loads ≥ 200 copies/mL, were documented in 4.4% of patients (37/830), with 10.9% occurrences (25/230) in the PIs group and 2.0% (12/600) in the INSTIs group (P < 0.001). The Kaplan-Meier estimates are shown in Fig. 2. Compared to the INSTIs group, the hazard ratio (HR) for the PIs group was 2.609 (95% CI: 1.057 to 6.438) (Fig. 2). Similarly, VF events characterized by viral loads ≥ 1000 copies/mL were observed in 3.4% of patients (28/830), comprising 8.3% cases (19/230) in the PIs cohort and 1.5% (9/600) in the INSTIs cohort (P < 0.001).

The occurrence of every LLV event on the risk of VF (defined as 50–199 copies/ml) was performed by the GEE model. The INSTIs-based regimen was the main protective factor, accounting for 76.9% (95% CI 59.1%-86.9%) of the declined risk in the incidence of VF (*P* < 0.001). However, the patients who experienced LLV during follow-up significantly increased 123.5% (95% CI 7.5%-364.4%) of the risk in VF incidence (*P* = 0.031) (Table 4). Similar results were obtained when VF was defined as ≥ 1000 copies/mL (Table 5).

**Table 4:** The GEE model for LLV events (defined as 50–199 copies/ml) of developing VF Events (defined as ≥ 200 copies/ml)

Risk factors	aHR (95% CI)	Increasing in risk, % (95% CI)	*P-value*
Male (vs. Female)	0.395 (0.168, 0.928)	‒60.5 (‒83.2, ‒7.2)	0.033
Age > 25 (vs. < 25 years)	1.634 (0.927, 2.882)	63.4 (‒7.3, 188.2)	0.090
Homosexual Transmission (vs. other)	1.664 (0.79, 3.505)	66.4 (‒21, 250.5)	0.180
Time from diagnosis to treatment ≤ 1 (vs. > 1 months)	1.221 (0.744, 2.002)	22.1 (‒25.6, 100.2)	0.429
CD4 + T cell count ≤ 200 (vs. > 200 cells/µL)	1.108 (0.629, 1.952)	10.8 (‒37.1, 95.2)	0.723
Viral load at baseline > 100,000 (vs. ≤ 100,000 copies/mL	1.312 (0.714, 2.408)	31.2 (‒28.6, 140.8)	0.381
LLV events during follow-up. (Yes vs. No) ^a^	2.235 (1.075, 4.644)	123.5 (7.5, 364.4)	0.031
INSTIs-based regimen (vs. PIs-based regimen)	0.231 (0.131, 0.409)	‒76.9 (‒86.9, ‒59.1)	< 0.001

a. The LLV events during follow-up were analyzed as the repeated measured variable

**Table 5:** The GEE model for LLV events (defined as 50–999 copies/ml) of developing VF events (defined as ≥ 1000 copies/ml)

Risk factors	aHR (95% CI)	Increasing in risk, % (95% CI)	*P-value*
Male (vs. Female)	0.318 (0.091, 1.106)	‒68.2 (‒90.9, 10.6)	0.072
Age > 25 (vs. < 25 years)	2.391 (0.877, 6.519)	139.1 (‒12.3, 551.9)	0.088
Homosexual Transmission (vs. other)	2.164 (0.685, 6.834)	116.4 (‒31.5, 583.4)	0.188
Time from diagnosis to treatment ≤ 1 (vs. > 1 months)	1.273 (0.5, 3.243)	27.3 (‒50, 224.3)	0.612
CD4 + T cell count at baseline ≤ 200 (vs. > 200 cells/µL)	1.448 (0.463, 4.526)	44.8 (‒53.7, 352.6)	0.525
Viral load at baseline > 100,000 (vs. ≤ 100,000 copies/mL	1.101 (0.282, 4.296)	10.1 (‒71.8, 329.6)	0.890
LLV events during follow-up. (Yes vs. No) ^a^	3.215 (1.097, 9.421)	221.5 (9.7, 842.1)	0.033
INSTIs-based regimen (vs. PIs-based regimen)	0.221 (0.084, 0.584)	‒77.9 (‒91.6, ‒41.6)	0.002

a. The LLV events during follow-up were analyzed as the repeated measured variable

Figure 2 legend: The X-axis represented the time since initiation of treatment, while the Y-axis represented the probability of maintaining viral suppression without experiencing virological failure. Censoring is indicated by small vertical marks along the survival curves.

## Discussion

In the era of integrase inhibitors, antiretroviral therapy has achieved unprecedented virological suppression in HIV-infected individuals. However, LLV persisted in patients under effective ART, which posed questions about the efficacy of current regimens in eradicating viral reservoirs and raised concerns about potential drug resistance. Our study indicates a lower incidence rate of LLV and VF among HIV populations in the era of integrase inhibitors. Notably, LLV was associated with an increase in the risk of subsequent treatment failure, while the use of integrase inhibitors was the main protective factor to mitigate this risk. These findings suggest that integrase inhibitors are effective in reducing VF risk but are not entirely successful in eliminating LLV. The persistence of LLV, despite reduced VF risk, highlights the need for further research to optimize treatment strategies.

The definition of low-level viremia in HIV research is not uniform and has evolved, reflecting advancements in viral load detection technologies and the changing thresholds for antiviral treatment failure in various treatment guidelines [23]. According to the constraints of middle and low-income countries, the WHO has set a viral suppression threshold at 1000 copies/mL [22]. Conversely, the Department of Health and Human Services (DHHS) and the International Antiviral Society (IAS) define virological suppression at a lower threshold of 200 copies/mL [19, 20]. Given these varying standards, the definition of LLV predominantly falls within two ranges: 50–199 copies/mL and 50–999 copies/mL. Moreover, there is a growing recognition of an even lower category termed ‘very low-level viremia’ (VLLV), defined as a viral load ranging from 20 to 49 copies/mL.

Our study employed two commonly used approaches for analysis: the 50–199 copies/mL and the 50–999 copies/mL ranges, which were analyzed in conjunction. The study’s findings reveal a markedly lower incidence of LLV in ART-naive patients on INSTIs-based regimens, compared to those on protease inhibitor regimens. This significant reduction in LLV underscored the enhanced virological efficacy of integrase inhibitors to achieve complete viral suppression compared to previous regimens (PIs) and supports their preferential use in first-line ART. However, the persistence of LLV, although at a lower rate, revealed that integrase inhibitors are not entirely successful in achieving complete viral eradication [24]. Understanding LLV in this context is pivotal for optimizing therapeutic strategies, as it may serve as a harbinger for treatment failure, drug resistance [25, 26], and disease progression. Clinicians should, therefore, remain vigilant in monitoring viral loads for patients exhibiting LLV, even when on integrase inhibitors [8].

Our study significantly contributes to the understanding of LLV in HIV treatment, particularly its association with VF. We found that LLV increases the risk of VF which was consistent with our previous research that identifies LLV as a critical factor in treatment outcomes [17]. Research in individuals with LLV highlighted the potential for ongoing inflammation, immune activation [27], increased risk of virologic failure [28], drug resistance [29, 30], non-AIDS-related complications [31, 32], and all-cause mortality [33]. This underscores the necessity for stringent virological monitoring to detect LLV early and adjust treatment regimens accordingly [13, 34]. However, the cost implications of rigorous virological testing can be prohibitive, especially in resource-limited settings. This financial constraint accentuates the significance of effective treatment strategies aimed at suppressing LLV. Remarkably, the integrase inhibitors were associated with a reduction in VF risk in our study. This finding is pivotal for the clinical management of LLV and suggests that integrase inhibitors should be considered as a first-line therapeutic option for patients experiencing LLV. Therefore, our results advocate for a targeted approach to treating LLV to prevent VF, thereby optimizing long-term patient outcomes.

Across all analyses, the lower baseline CD4 cell counts and higher baseline viral loads are significantly associated with increased risks of both LLV and VF, emphasizing the need for early diagnosis and immediate ART [19]. The advent of immediate ART has revolutionized HIV management, offered the promise of sustained viral suppression and improved long-term outcomes [35–39]. However, the phenomenon of LLV poses a clinical conundrum in this era of early treatment. While Immediate ART initiation aims to preserve immune function and lower set-point viral loads [38], the occurrence of LLV suggests incomplete viral suppression. Further research is imperative to elucidate the long-term clinical implications of LLV in the context of immediate ART initiation. Our study did not identify a significant association between gender and LLV, which may be attributed to the overrepresentation of male-to-male transmission and the underrepresentation of heterosexual populations in our study sample. Thus, further research is required to comprehensively validate these findings.

There were limitations in this study. First, our study was a cohort study and presented uneven baseline characteristics between the two groups. Therefore, we applied multivariate analysis to adjust the baseline characteristics. Secondly, the limited occurrence of antiretroviral treatment failure outcomes with integrase inhibitor regimens hampered the precise assessment of differences among the BIC, DTG, RAL, and EVG regimens. Thirdly, the single-center cohort restricts generalizability. These constraints underscore the complexity of our findings and underscore the necessity for more extensive and diverse cohorts to comprehensively address these research gaps.

In conclusion, the findings of this study suggest that the occurrence of low-level viremia in HIV patients is closely linked to virological failure. INSTIs-based regimens are critical protective factors against the occurrence of low-level viremia during integrase inhibitor therapy and subsequent prevention of virological failure. However, the present research still faced certain challenges, including the need for a more comprehensive understanding of optimal intervention timing, long-term consequences of low-level viremia, and potential drug resistance concerns. Addressing these research gaps is essential to refining HIV treatment strategies and bolstering the effectiveness of integrase inhibitor-based therapies.

## Data Availability

The datasets used and/or analysed during the current study are available from the corresponding author on reasonable request.

## Abbreviations

ART: Antiretroviral therapy
GEE: Generalized Estimating Equation
HIV: Human immunodeficiency virus
IQR: Interquartile ranges
INSTIs: Integrase Strand Transfer Inhibitors
LLV: Low-level viremia
NFATP: Chinese National Free Antiretroviral Treatment Program
PIs: Protease inhibitors
PLWH: People living with HIV
VL: Viral load
VF: Virologic failure
